# Trabecular micro-bypass implant (iStent®) in a case of bilateral acute depigmentation of the iris

**DOI:** 10.5935/0004-2749.2021-0239

**Published:** 2022-09-06

**Authors:** Heloisa Andrade Maestrini, Angela Andrade Maestrini, Sarah Pereira de Freitas Cenachi, José Aloisio Massote, Amanda Batista Lopes, Thatiana Almeida Pereira Fernandes

**Affiliations:** 1 Department of Glaucoma, Oculare Hospital de Oftalmologia, Belo Horizonte, MG, Brazil; 2 Department of Uveitis, Oculare Hospital de Oftalmologia, Belo Horizonte, MG, Brazil

**Keywords:** Iris disease, Cataract, Ocular hypertension, Stents, Gonioscopy, Doenças da íris, Catarata, Hipertensão ocular, Stents, Gonioscopia

## Abstract

We report a case of bilateral acute depigmentation of the iris in which satisfactory intraocular pressure control was obtained after resolution of the acute disease with a trabecular implant (iStent®). A 62-year-old woman presented with bilateral simultaneous acute eye pain, photophobia, increased intraocular pressure (34 mmHg), circulating pigment in the anterior chamber, areas of depigmentation in the iris, and posterior synechiae. She had received oral amoxicillin-clavulanate and moxifloxacin for pneumonia 2 months previously. Bilateral acute depigmentation of the iris was suspected as well as a viral etiology. She received oral acetazolamide, aciclovir, and prednisone, besides topical prednisolone, betaxolol, brimonidine, dorzolamide, and atropine. The disease gradually resolved in 4 months but, after 1 year, she developed bilateral cataracts, and still needed three drugs for intraocular pressure control (16/18 mmHg). Cataract-iStent® combined surgery was performed in both eyes. One year after surgery, intraocular pressure was 11/12 mmHg, without medication. iStent® was safe and effective on this secondary glaucoma.

## Abstract (pt)

Relatamos um caso de despigmentação aguda bilateral da íris, no qual obtivemos adequado controle da pressão intraocular com o implante do iStent®, após resolução da fase aguda da doença. Paciente feminina, 62 anos, atendida com quadro agudo, bilateral e simultâneo de dor ocular, fotofobia, hipertensão ocular (34 mmHg), pigmentos circulantes na câmara anterior, áreas de despigmentação iriana e sinéquias posteriores. Havia recebido amoxicilina-clavulanato e moxifloxacina orais para pneumonia 2 meses antes. Suspeitando-se de despigmentação aguda bilateral da íris ou de etiologia viral, recebeu acetazolamida, aciclovir e prednisona orais, e colírios prednisolona, betaxolol, brimonidina, dorzolamida e atropina. O quadro se resolveu gradualmente em 4 meses, porém, após 1 ano, desenvolveu catarata bilateral e ainda usava 3 colírios hipotensores (pressão intraocular 16/18 mmHg). A cirurgia combinada de catarata-iStent® foi realizada em ambos os olhos. Um ano depois, a pressão intraocular mantinha-se 11/12 mmHg, sem medicação. O iStent® foi seguro e eficaz no controle deste glaucoma secundário.

## INTRODUCTION

Bilateral acute depigmentation of the iris (BADI) was first described by Tugal-Tutkun in 2006^(1)^ and is characterized by sudden onset of bilateral irregular depigmentation of the iris, intense pigment dispersion into the anterior chamber (AC), and heavy pigment deposition in the trabecular meshwork, leading to trabecular obstruction and consequent rapid elevation of intraocular pressure (IOP). The first case in Brazil was reported in 2013^(2)^. BADI is more commonly seen in middle-aged women, and is always bilateral, simultaneous, and often symmetrical. The main symptoms are conjunctival hyperemia, photophobia, ocular pain, and blurred vision. The etiology remains unclear. Upper respiratory tract infections (URTI) have often been described before the onset of BADI, which some authors have suggested may be triggers of the syndrome^(3)^. Significant numbers of these cases had been treated with oral moxifloxacin, suggesting that it may play a role in the etiopathogenesis of the syndrome^(4)^ due to potential toxicity to the iris pigment epithelium^(5)^. In addition, due to similarities with some types of viral iridocyclitis, especially those related to the herpes family, a viral etiology has been hypothesized^(6)^. BADI shares many common features with bilateral acute iris transillumination (BAIT)^(6)^, with the difference that BAIT is associated with massive transillumination defects, dilated and nonreactive pupils, posterior synechiae, and higher IOP levels. In fact, BADI and BAIT could be different aspects of the same disease. Kawali et al. reported cases in which one eye exhibited features of BADI, whereas the other exhibited features of BAIT, confirming the relationship between the two syndromes^(5)^. Clinical signs and symptoms usually subside after a few weeks or months, but ocular hypertension may be persistent. Here, we describe a case of ocular hypertension after BADI treated successfully with iStent® implantation after of the resolution of the acute phase.

## CASE REPORT

**Figure 1:**
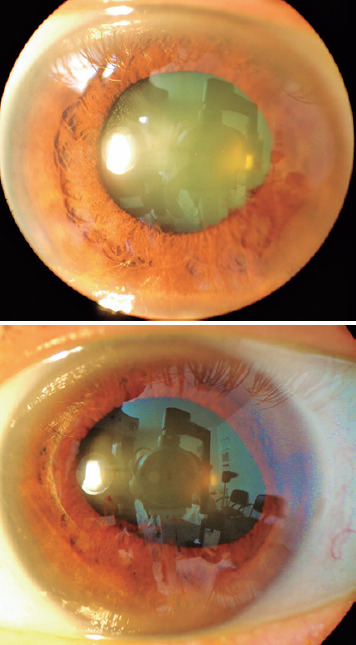
Biomicroscopy OD/OS. Dilated and distorted pupils with small posterior synechiae and the mid-periphery areas of depigmentation in the iris.

A 62-year-old woman was first evaluated in April 2019 with acute onset of bilateral eye pain and photophobia. Visual acuity was 20/20 in both eyes. Examination revealed bilateral involvement with ciliary injection, circulating pigment in the AC, patchy areas of iris depigmentation, posterior synechiae, and ocular hypertension (34 mmHg). Pupils were distorted, sluggish, and slightly dilated, but there were no iris transillumination defects (Figure 1). There were also no inflammatory keratic precipitates. The vitreous was clear, and the fundus and optic discs were normal (C/D ratio 0.4/0.5). She had a history of pneumonia and sinusitis two months earlier, which had been treated with oral amoxicillin-clavulanate and moxifloxacin.

**Figure 2:**
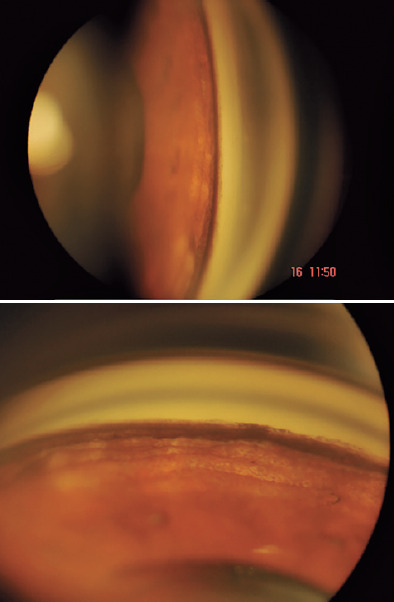
Gonioscopy views of the temporal and inferior regions showing a heavily pigmented angle and depigmentation of the iris at the periphery.

**Figure 3:**
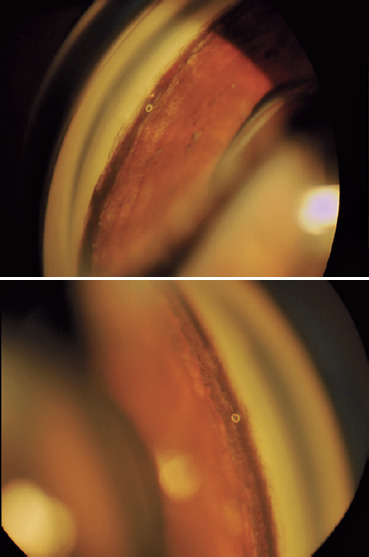
Postoperative gonioscopy views showing the iStent® aspect. OD/OS.

At presentation, she was examined by a uveitis specialist and a rheumatologist, and underwent an extensive laboratory workup to search for rheumatological and infectious causes. All results were normal, and no specific diagnosis was established. Nevertheless, she had IgG antibodies for the herpes simplex virus, varicellazoster virus, and cytomegalovirus. BADI was suspected, as well as an unusual presentation of bilateral herpetic uveitis. She received oral aciclovir, acetazolamide, and prednisone, besides topical prednisolone, betaxolol, brimonidine, dorzolamide, and atropine bilaterally. After two months, topical corticosteroid was withdrawn, with resumption of symptoms. Prednisolone eye drops were reintroduced and gradually removed over the next two months, as well as all oral medications, with gradual resolution of the disease. Due to persistent ocular hypertension, she was referred to our Glaucoma Department in August 2019. Gonioscopy revealed, bilaterally, a wide and heavily pigmented angle 360°, and a gross pigment deposit in the inferior angle (Figure 2). On fundus examination, the optic discs were normal, with no signs of glaucomatous neuropathy. The IOP was controlled (16/18 mmHg) with three drugs (betaxolol, brimonidine, and dorzolamide). One year after the diagnosis, in April 2020, she developed bilateral cataracts. Combined cataract-iStent® surgery was proposed for both eyes and performed in May and June 2020. The purpose of iStent® implantation was to reduce at least one glaucoma medication. Surprisingly, it was possible to remove all hypotensive agents. At one year after surgery, the IOP was 11/12 mmHg without medication (Figure 3) and visual acuity was 20/15 in both eyes.

## DISCUSSION

Our patient had typical BADI with sudden onset of bilateral irregular depigmentation of the iris, without transillumination defects, intense pigment dispersion into the AC, heavy pigment deposition in the trabecular meshwork, and ocular hypertension, but with some features of BAIT (posterior synechiae and dilated, distorted, and sluggish pupils). As stated in the Introduction, there is a probable etiopathogenic relationship between the two syndromes^(6)^. As commonly reported in both entities, she had a history of previous upper respiratory tract infection that had been treated with oral moxifloxacin. The acute phase resolved in four months, but ocular hypertension persisted.

The diagnosis of BADI is based on clinical findings. The main differential diagnosis is bilateral iridocyclitis, especially herpetic, idiopathic, and Fuchs. In BADI, no inflammatory cells or keratic precipitates are seen. AC tap for polymerase chain reaction analysis can be performed to rule out viral infections, but it may be insensitive in the absence of posterior segment involvement. BADI also must be differentiated from other chronic pigment conditions such as pigment dispersion syndrome and pseudoexfoliation.

The iStent® was primarily developed to treat open-angle glaucoma^(7)^, especially when combined with phacoe-mulsification. Nonetheless, it can also be used in some secondary glaucomas, such as exfoliative, pigmentary, corticoid-induced, and even traumatic glaucomas^(8,9)^. These reports prompted us to apply the iStent® in the present case.

Pigment deposition initially affects the trabecular meshwork but can lead to damage in the post-trabecular drainage system after some time. Following this rationale, if we bypass this obstruction in the early phase, it may be possible to restore the entire trabecular pathway^(10)^, thus preventing post-trabecular damage. This can explain the excellent results with iStent® implantation in the present case, suggesting that the post-trabecular drainage system was viable, possibly due to the small amount of time elapsed since the onset of the disease (only 1 year). In conclusion, iStent® proved to be effective and safe to control IOP in this case of BADI, after resolution of the acute phase of the disease. This is the first report of iStent® use on a case of BADI.
